# Livelihoods and Fisheries Governance in a Contemporary Pacific Island Setting

**DOI:** 10.1371/journal.pone.0143516

**Published:** 2015-11-23

**Authors:** Reuben J. Sulu, Hampus Eriksson, Anne-Maree Schwarz, Neil L. Andrew, Grace Orirana, Meshach Sukulu, Janet Oeta, Daykin Harohau, Stephen Sibiti, Andrew Toritela, Douglas Beare

**Affiliations:** 1 WorldFish, Honiara, Solomon Islands; 2 WorldFish, Penang, Malaysia; 3 Australian National Centre for Ocean Resources and Security (ANCORS), University of Wollongong, Wollongong, Australia; 4 Malaita Provincial Fisheries Division, Malaita Province, Solomon Islands; Leibniz Center for Tropical Marine Ecology, GERMANY

## Abstract

Inshore marine resources play an important role in the livelihoods of Pacific Island coastal communities. However, such reliance can be detrimental to inshore marine ecosystems. Understanding the livelihoods of coastal communities is important for devising relevant and effective fisheries management strategies. Semi-structured household interviews were conducted with householders in Langalanga Lagoon, Solomon Islands, to understand household livelihoods and resource governance in fishing-dependent communities. Households were engaged in a diverse range of livelihoods. Fishing, shell money production and gardening were the most important livelihoods. Proximity to an urban centre influenced how households accessed some livelihoods. Perceptions of management rules varied and different reasons were cited for why rules were broken, the most common reason being to meet livelihood needs. Current models of inshore small-scale fisheries management that are based on the notion of community-based resource management may not work in locations where customary management systems are weak and livelihoods are heavily reliant on marine resources. An important step for fisheries management in such locations should include elucidating community priorities through participatory development planning, taking into consideration livelihoods as well as governance and development aspirations.

## Introduction

Inshore small-scale fisheries play a significant role in the livelihoods of coastal communities in many developing countries both for food and income generation [1,2]. The level of dependence varies among locations and is affected by factors such as: the availability of cultivable land [3–6]; access rights and rules governed by cultural institutions [7]; local social dynamics [8]; local demographic dynamics [9] and access to markets [10–12]. In many locations, fish provides the majority of dietary animal protein needed for subsistence [13]. Such a high reliance on fish can result in damage to coral reef ecosystems from overfishing [14,15] especially in areas with a high-density human population with few or no livelihood alternatives [11]. In this context, environmental and ecological considerations necessarily become secondary to the imperative to feed families [16], and such complexities must be understood in order to guide appropriate strategies for fisheries management [12,17].

Customary marine tenure systems have been described as an important basis for inshore fisheries management in the Pacific [18]. However it is becoming clear that customary management systems alone are not effective in meeting community fisheries management goals and will require re-imagining and hybridisation with modern methods of fisheries management [19]. Community-based resource management (CBRM) approaches dominate contemporary strategies for inshore fisheries management in the Pacific [20]. They are widely adopted by non-governmental organisations (NGOs) in the region, largely because of the predominance of traditional management systems, poor formal fisheries governance structures [21] and ineffective central agencies [22,23]. While there are some documented examples of successful management outcomes [17,23,24] there is mixed evidence to date from Solomon Islands [20,25,26].

The CBRM model builds on the strength of empowered communities emanating from governance reforms by which governments allow organised communities to shape the institutions that coordinate and manage resource use [27,28]. The premise is that these new institutions are more effective when led by the resource users themselves. This study examined livelihood considerations within fisheries governance in a contemporary Pacific Island setting. The aim is to understand how livelihoods within a highly fishery-dependent population in which marine resources are contested due to high levels of competition between people can affect fisheries governance and CBRM.

## Langalanga Lagoon and Its People

The study was conducted in Langalanga Lagoon, Malaita Province, Solomon Islands. Malaita has the lowest human development index of all the provinces in the country. It is also the most populous (150,000), comprising 27% of the total Solomon Islands population [29]. The people in the area are highly dependent on marine resources, and there are few realistic alternative livelihood options [30]. Langalanga Lagoon is close to the semi-urban centre of Auki, the provincial capital. It is also an important commercial hub where people from Langalanga and other nearby parts of Malaita sell their primary produce (fish, shellfish and agricultural products).

**Fig 1:**
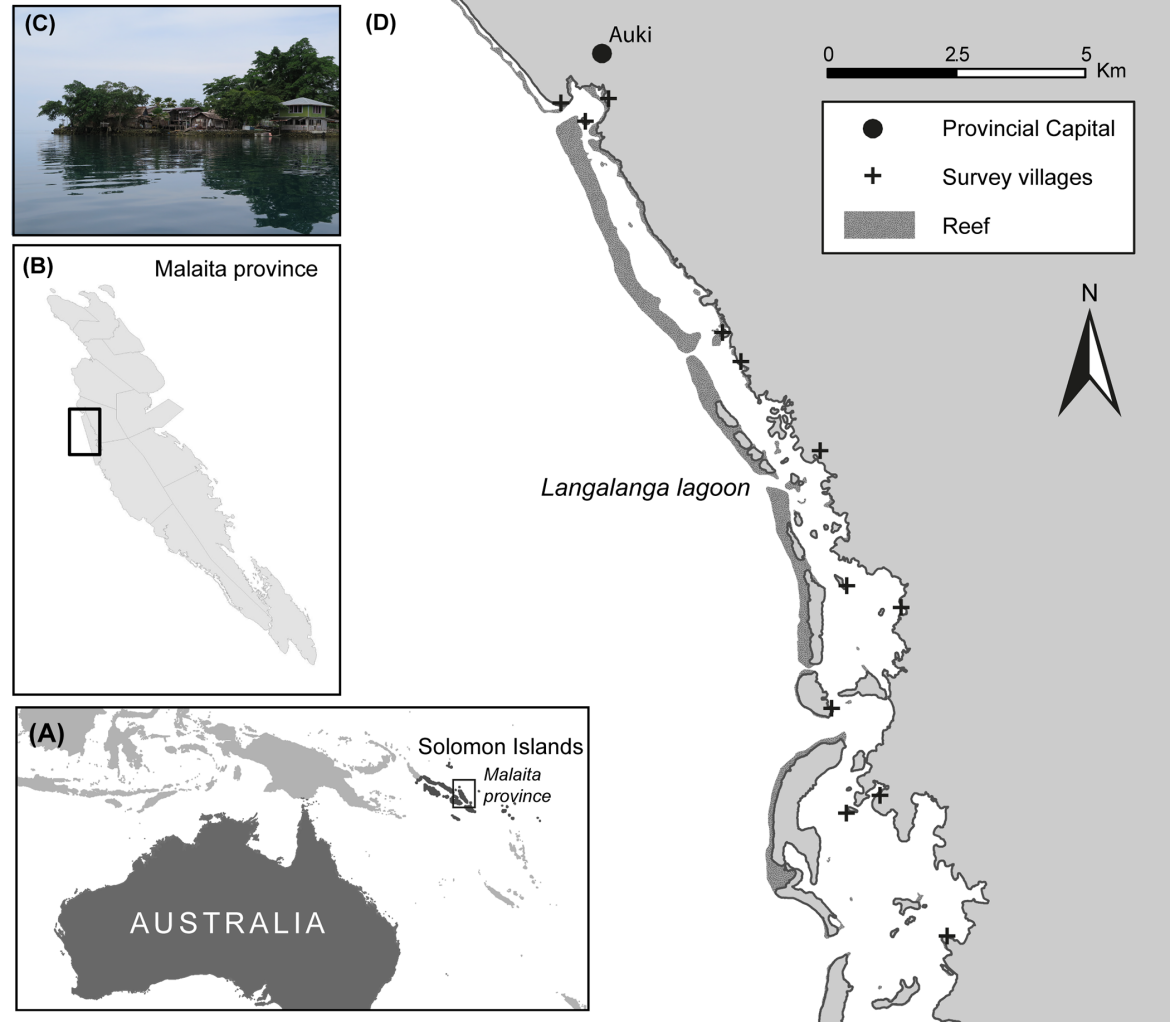
Map of Langalanga Lagoon, Solomon Islands. (A) Solomon Islands are located in the Western Pacific. (B) Langalanga lagoon is situated on the west coast of Malaita Province. (C) Many communities in the lagoon live on the high-water mark on artificial islands. (D) The lagoon extends south of the provincial capital Auki; twelve communities were included in the study.

The people of Langalanga Lagoon are confined to a very thin strip of coastal area with limited access to land for crop cultivation according to customary law (Fig 1), a marked contrast to the neighbouring mainland ‘bush people’ who have access to comparatively abundant agricultural land. Langalanga people live either on artificial islands just off the mainland or on the coastal fringes of the mainland [31]. Although they do partly depend on agricultural production [32], their livelihoods have mostly revolved around the sea for several hundred years. Indeed, the people of Langalanga Lagoon are referred to (and proudly refer to themselves) as the *solwata pipol* (‘saltwater people’). Their involvement in agricultural food production has declined in recent years, resulting in an increasing need to purchase a substantial component of their food from the ‘bush people’ or buy imported items from shops [33]. According to Faradatolo [34], food for household consumption comprises 65% of total household expenses in Langalanga; a contrast to Solomon Islands in general, where subsistence gardening and fishing are the predominant contributors to daily consumption [35]. An increased reliance on store-bought foods not only influences livelihood strategies through the need for cash, but these foods are often high in salt, sugar and fat—with associated implications for health [36].

At the time of study, the total population of Langalanga Lagoon was about 16,500 people [29]. Despite this relatively high population density and proximity to Auki, there is no centralised electricity supply beyond the immediate environs of Auki and people largely rely on mangrove wood for cooking over open fires [37]. A limited number of households have solar power for lighting and bottled gas for cooking. Water is mostly drawn from wells or rain-fed aluminium water tanks [30], as very few villages have access to piped water from mainland water sources. Sanitation is generally lacking, with coastline areas, such as mangroves or over-water latrines, serving sanitation purposes [38].

With an increasing population, demand for cash and destructive fishing practices, the people of Langalanga Lagoon, like many other Pacific Islands, are increasingly faced with issues around resource governance [39]. Langalanga Lagoon has been listed internationally as a site with severe blast-fishing activities [40], and dynamite fishing is used on a daily basis [38]. Mangrove removal for establishment of human settlement and extraction for firewood and building materials is a common occurrence in Langalanga [37,38]. The same holds true for the extraction of massive *Porites* corals for the construction of artificial islands or land extensions [30,38], and *Acropora* corals for the production of quicklime used in the consumption of a traditional narcotic palm seed—betelnut (*Areca catechu*).

The customary management systems are typically weak [41] and this has in part hampered various attempts by resource owners in Malaita Province in general to institute rules, or stimulate changes in behaviour around resource use [42]. In order to devise relevant fisheries management strategies for Langalanga Lagoon, an in-depth understanding of the drivers of patterns of resource use for livelihood purposes and their perceived impacts on resources is required [12,16]. Responding to the interest expressed by many lagoon dwellers and initiatives undertaken by some in finding ways to effectively manage their marine resources, this study analysed the range and complexity of livelihood strategies adopted by the people of Langalanga Lagoon, and how they are connected with fisheries management. The implications for coastal resource management approaches, including co-management models in Solomon Islands and beyond, are discussed.

## Materials and Methods

### 3.1 Ethical statement

The research was approved by the WorldFish project management system. Further ethical clearance was provided through a Memorandum of Understanding between WorldFish and the Solomon Islands Government and by the WorldFish Code of Ethics for working with people (2009). Interviewees gave verbal consent to participate in the study and if verbal consent was not given the interview did not proceed. Written consent was not sought because of low levels of literacy. WorldFish approved the verbal consent process. Village names are not provided to maintain community confidentiality.

### 3.2 Field methods

Household socio-economic surveys using a structured questionnaire were conducted in 12 villages (see Fig 1 for locations) in Langalanga Lagoon in March 2013, following the approaches of Cinner [43], Swindale and Bilinsky [44] and Coates et al. [45] for different sections as relevant. The survey team comprised exclusively Solomon Islanders (4 males and 2 females) who were familiar with the culture and fluent in pijin (a lingua franca in Solomon Islands). Two members of the survey team were from Langalanga and could also speak the local language. Respondents were usually heads of households, including both males and females. In many cases, both the heads of the households and some of their children were present for the interview. When more than one person in the household was present, other family members also contributed with remarks to either support or correct those made by the heads of households.

In each village, 30% of the households were surveyed. Depending on the number of houses, the *n* ^th^ house was selected sequentially starting from one end of the village. Where people were not available in the selected household, then the people in the next house were interviewed. The number of households to be interviewed in a particular village was determined prior to the survey using census data from the Solomon Islands National Statistics Office. For small villages (particularly those on small artificial islands), with usually 10 or fewer houses, all households were interviewed.

A total of 235 households were interviewed (about 10% of all households in the lagoon). Respondent were asked questions about: (i) household information; (ii) household economics which included gendered roles that contribute to food or income to the household; (ii) dietary diversity and food insecurity, and (iv) access to natural resources.

The questions on household information and economics elicited data on: household demographics; livelihood activities undertaken to obtain food or generate income; who performs what activities; importance ranking for the different livelihood activities (where 1 = most important, 2 = important, 3 = moderately important and 4 = least important); whether the particular activity produced food, generated income or both; and the mean income typically generated from each livelihood activity. Questions on dietary diversity followed the methods described by Swindale and Bilinsky [44], in which interviewees were asked about the types of food consumed by a household in the 24 hours prior to the interview. Questions on food insecurity followed the methods of Coates et al. [45], in which the information sought was, in the 4 weeks prior to the survey, whether households were: worried about availability of food; had food available; had enough food to eat; and were eating the foods that they preferred. If the response was that households were worried about their food supply, food was sometimes not available, or they were eating foods that were not preferred, then a further question was asked as to how often this occurred (where 1 = rarely: 1–2 times in the 4 weeks prior to the survey, 2 = sometimes: 3–10 times in that 4 weeks, or 3 = often: more than 10 times).

Access to natural resources was compared among householders by asking who had access to fishing opportunities, what determined this access, whether there were rules governing access and use, and whether the rules were followed or not and if not then why not? Governance-related questions asked whether there were leaders in the community, how the leaders were chosen and whether the leaders were respected. Interviewers also took notes from any ensuing discussions during the interviews, and these notes—along with key informant interviews—served as qualitative data used to complement the quantitative survey data.

### 3.3 Data analysis methods

The data were entered into a Microsoft Access 2010 database. Data analysis was completed in R version 3.0.2 [46].

To test the hypothesis that distance to the urban centre and access to agricultural land for gardening played a role in shaping livelihood strategies, household economics, the type of livelihoods, access to natural resources and dietary diversity, all data were examined in relation to distance of the villages from Auki, and whether villages were on artificial islands or the mainland. Livelihood diversity patterns between island and mainland households were compared using a chi-square test. The relationship between weekly income, livelihood diversity and household food insecurity scores were examined using Kendall’s tau-b correlation coefficient for non-parametric data.

Following Mills et al. [47], we adopted a weighting approach to determine relative importance of livelihoods. To determine the relative importance of activities, each importance ranking was converted to a score, where rank 1 = 4 points and rank 4 = 1 point. Each livelihood activity was then weighted according to the frequency it was mentioned in a particular village as per the formula (Eq 1):

$$Li=\left(\frac{\sum_{r=1}^{4}lr}{nl}\right)\cdot \left(\frac{nl}{N}\right)$$ (1)

Where *Li* = weighted mean livelihood importance score for a particular livelihood activity

*lr* = livelihood rank points for a particular livelihood activity

*nl* = the number of times (counts) a particular livelihood activity was mentioned in a particular village

*N* = total count of all livelihood activities mentioned in a particular village.

Weighted mean livelihood importance scores for the 6 most-common livelihood activities (fishing, shell money production, gardening, casual work, petty trading and remittances) were then correlated with distance from Auki using a standard linear regression model function available in R [46]. Differences between mainland and island scores were analysed using a Welch two sample t-test after testing for normality using Bartlett’s test.

We examined gender roles in livelihood activities by performing a multinomial logistic regression in R [46] to calculate the probability of involvement in the six most-common livelihood activities as a function of different types of household members and zones. Zones served as a proxy for increasing distance from Auki—each village was assigned to one of three arbitrarily defined, equal length zones (1 = close, 2 = middle, 3 = far).

The analysis of food diversity followed the methods of Swindale and Bilinsky [44] while analysis of food insecurity followed Coates et al. [45]. Food diversity was determined by grouping the different foods into 12 main categories (cereals (grains e.g. wheat flour or rice); roots and tubers; vegetables; mangrove propagules; fruits; meat, poultry and offal; eggs; fish and shellfish; pulses, legumes and nuts; dairy; oil and fats; sugar and honey): the maximum dietary diversity score was therefore 12. Food insecurity was determined by summing the frequency score of food insecurity as per Coates et al. [45]; the maximum possible score representing a more food-insecure household was 27, while the lowest possible score (less food-insecure) was 0. Food insecurity score comparison between mainland and island initially involved testing for normality of data using the Shapiro-Wilks test. The data were non-normal so the Fligner-Killen test was used to test for homogeneity of variance; since variance of data was non-homogenous, a non-parametric test (Wilcoxon-Mann Whitney U) was ultimately used to compare food insecurity scores between mainland and island.

In all, 530 responses about existing fisheries management rules and their level of compliance were recorded in the survey (note: each of the 235 respondents were allowed more than one response). Responses were first categorised by the type of authority seen to be instituting those rules (traditional belief systems, privately owned areas, national regulations (and misconceived regulations) and modern community regulations) and then further divided into five rule types (gear restrictions; species restrictions; spatial restrictions; size restrictions; and temporal restrictions) and four levels of compliance (all comply; most comply, few comply and no compliance). Spatial restrictions refer to areas which were permanently closed due to traditional beliefs or private protection, while temporal restrictions refer to areas which were normally closed but could be opened periodically. Compliance levels to different rule types were further quantified as a percent (%) of the total number of responses under a particular type of authority. Responses (*n* = 251) to the open-ended question ‘Why do people violate resource management rules?’ were subsequently grouped into nine categories based on similar themes.

## Results

### 4.1 Livelihood strategies

A household was generally described by the respondents as a group of people planning things together and eating the same meals. There were instances of more than one ‘household’ living in the same physical dwelling. Based on this definition, the household size ranged from 1 to 17, with a median of 6 persons per household.

**Fig 2:**
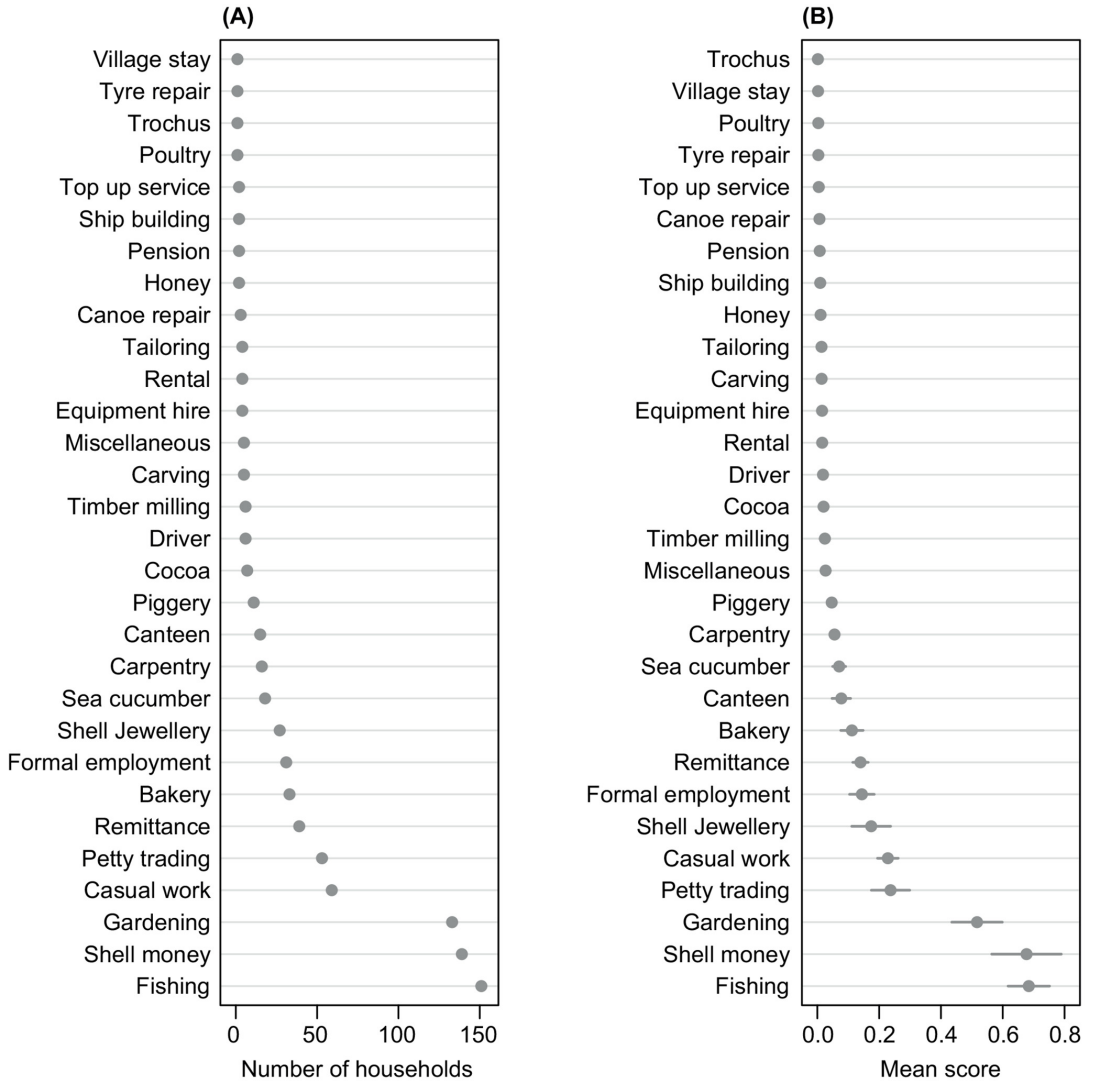
Household livelihood activities. (A) Frequency distribution of the number of households mentioning different livelihood activities and (B) weighted mean livelihood importance score (*Li*).

**Fig 3:**
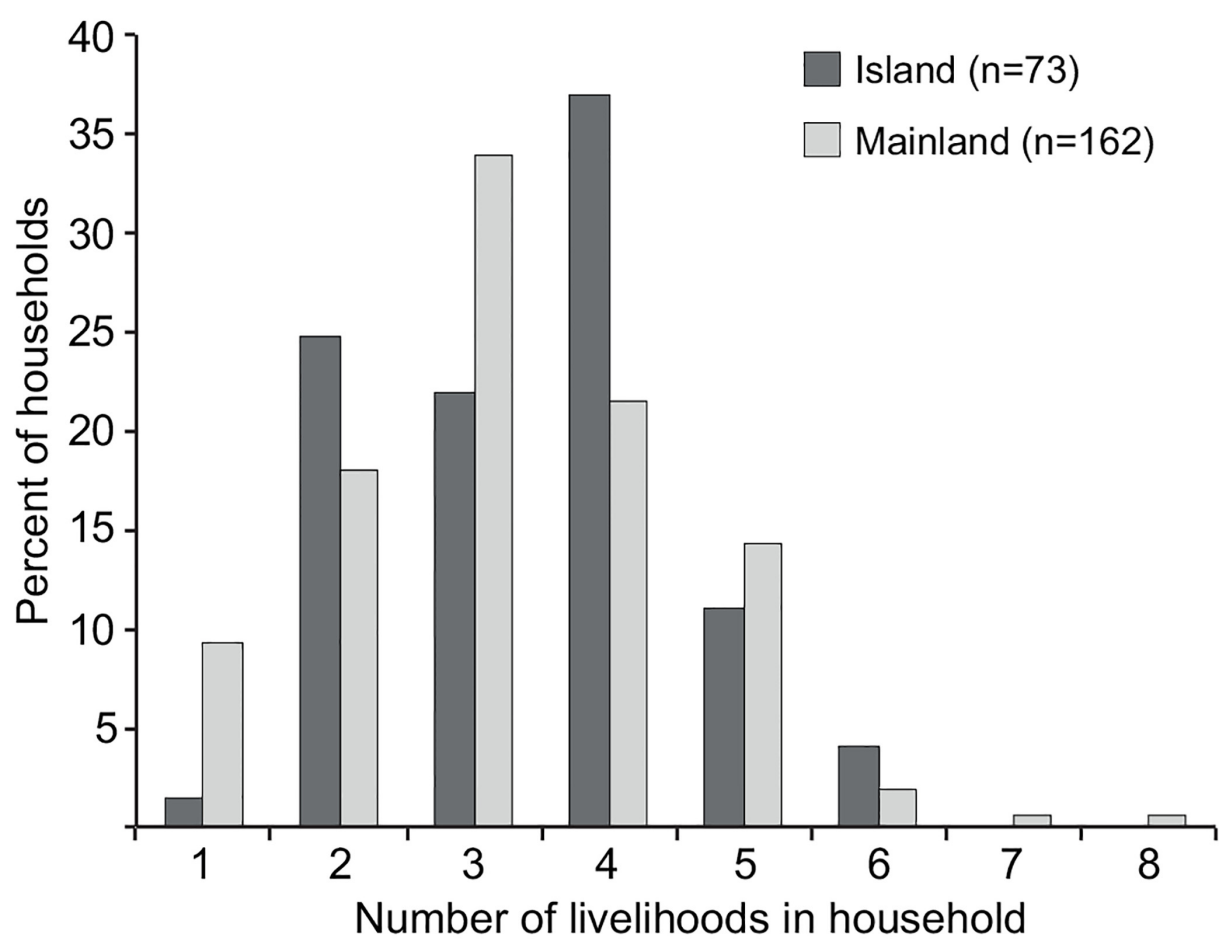
Livelihood diversity in Langalanga Lagoon. Percent of island and mainland households engaged in a number of livelihood activities.

All surveyed households were engaged in multiple livelihood activities, producing food and/or generating income. Thirty types of activities were noted; 7 were land-based, 6 were marine-based and 17 involved trading or were skills based (including paid casual or full time employment). Some activities were undertaken only for income, e.g. shell money (traditional form of currency used in cultural practices and exchanges) and shell jewellery production, while others both for food and cash, e.g. fishing, gardening and baking. The most common and highest scoring (*Li*) livelihood activities were fishing, shell money production and gardening (Fig 2). Other important activities were petty trading, casual work, shell jewellery production, formal employment, remittances (money sent from family members living away from households) and baking. All other activities scored 0.1 or less. Household livelihood diversity ranged from two to eight activities per household. The median livelihood diversity for island households was four, while for mainland households it was three. There was a significant difference (*X* ^*2*^ = 15, *df* = 7, *p* = 0.04) in number of livelihoods utilized by island and mainland households (Fig 3). There was no significant relationship between livelihood diversity and weekly income both for island (*tau-b* = -0.01, *p* > 0.05) or mainland households (*tau-b* = -0.06, *p* > 0.05).

**Fig 4:**
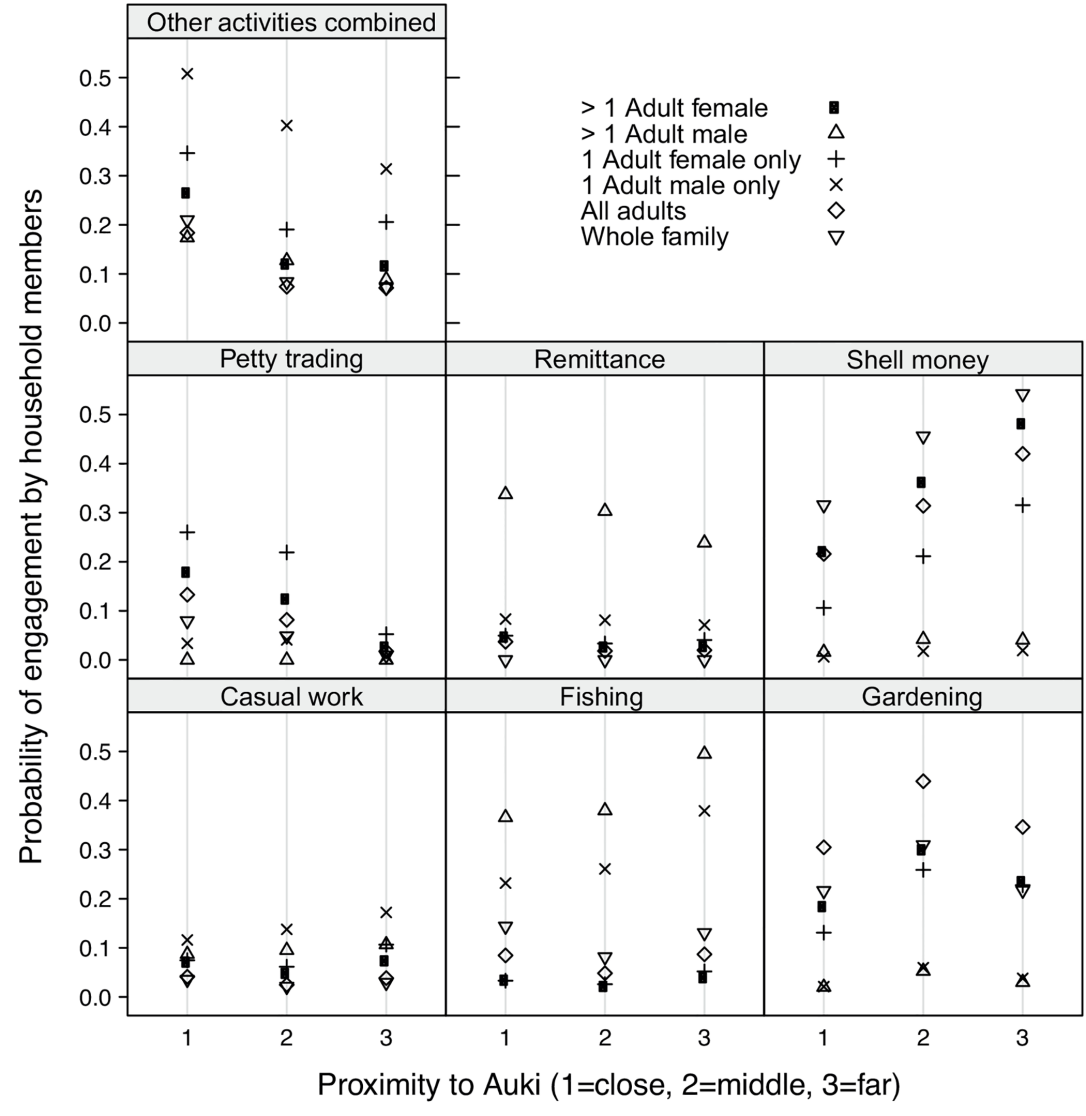
Distribution of livelihood activities across family members. Multinomial logistic regression plot of the probability of involvement in the six most common livelihood activities, and all other livelihood activities combined, as a function of household members and proximity to Auki.

Although both men and women were involved in all income-generating activities, there were some clear differences in gender roles (Fig 4). Remittance, casual work and fishing were mostly mentioned as income sources by adult males, although the data also suggested that women were increasingly becoming involved in inshore fishing. The production of shell money and shell jewellery was dominated by women, although both men and women had specific roles in this activity—breaking shells into smaller beads and drilling shells were women’s role, while filing and smoothing shell beads was done by men. Adult females were most commonly involved in petty trading and gardening, but all members of the household (including children) could be involved. For the combined activities aside from the most common six, the probability of one adult male only in the household participating was higher than for one adult female only (top single panel of Fig 4).

**Fig 5:**
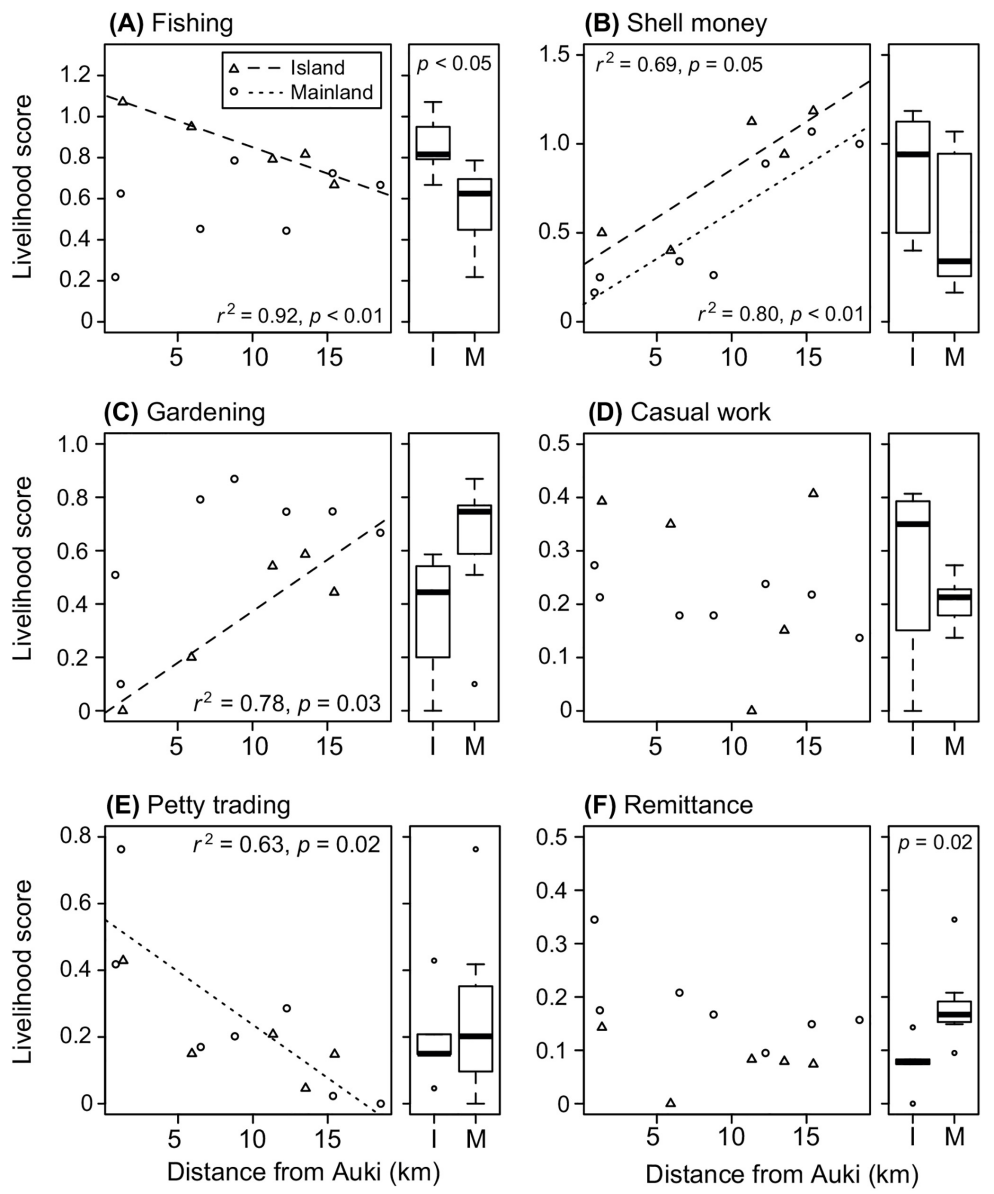
Significance of distance from Auki on livelihood activities. Relationship between weighted mean livelihood importance score (*Li*) and distance from Auki (km) for the six most-common livelihood activities. Differences in slopes were detected for fishing, shell money, gardening and petty trading (panels A, B, C and E). The Tukey’s boxplots illustrate the range, median and upper and lower quartiles of *Li* at island (I) and mainland (M) households for each of the six most common livelihood activities. Significant difference in *Li* between island and mainland households was found for fishing (panel A), and remittance (panel F).

The livelihood importance score (*Li*) for fishing showed a negative trend for island households with increasing distance from Auki (*r* ^2^ = 0.92, *p* < 0.01). Island households scored an overall higher *Li* for fishing than mainland households did (*t* = 2.93, *df* = 9.84, *p* = 0.01) (Fig 5A, boxplot). Shell money production became increasingly important further away from Auki to both island (*r* ^2^ = 0.69, *p* = 0.05) and mainland (*r* ^2^ = 0.80, *p* < 0.01) households (Fig 5B), supporting qualitative field observations that there was relatively little involvement in shell money production in villages near Auki compared to those further away. A similar pattern was evident for gardening in island households (*r* ^2^ = 0.78, *p* = 0.03) (Fig 5C), but not for mainland households. Out of the 133 households involved in gardening, 119 (89%) produced vegetables solely for domestic consumption. The importance of casual work showed no relationship with distance from Auki (Fig 5D). Petty trading scored higher in mainland households close to Auki than those further away (*r* ^2^ = 0.63, *p* = 0.02) (Fig 5E). Mainland households scored remittance (not an activity carried out at Langalanga but which contributes to livelihoods) higher than island households (*t* = -2.93, *df* = 9.97, *p* = 0.01) (Fig 5F, boxplot), but no trend with distance to Auki was detected.

**Fig 6:**
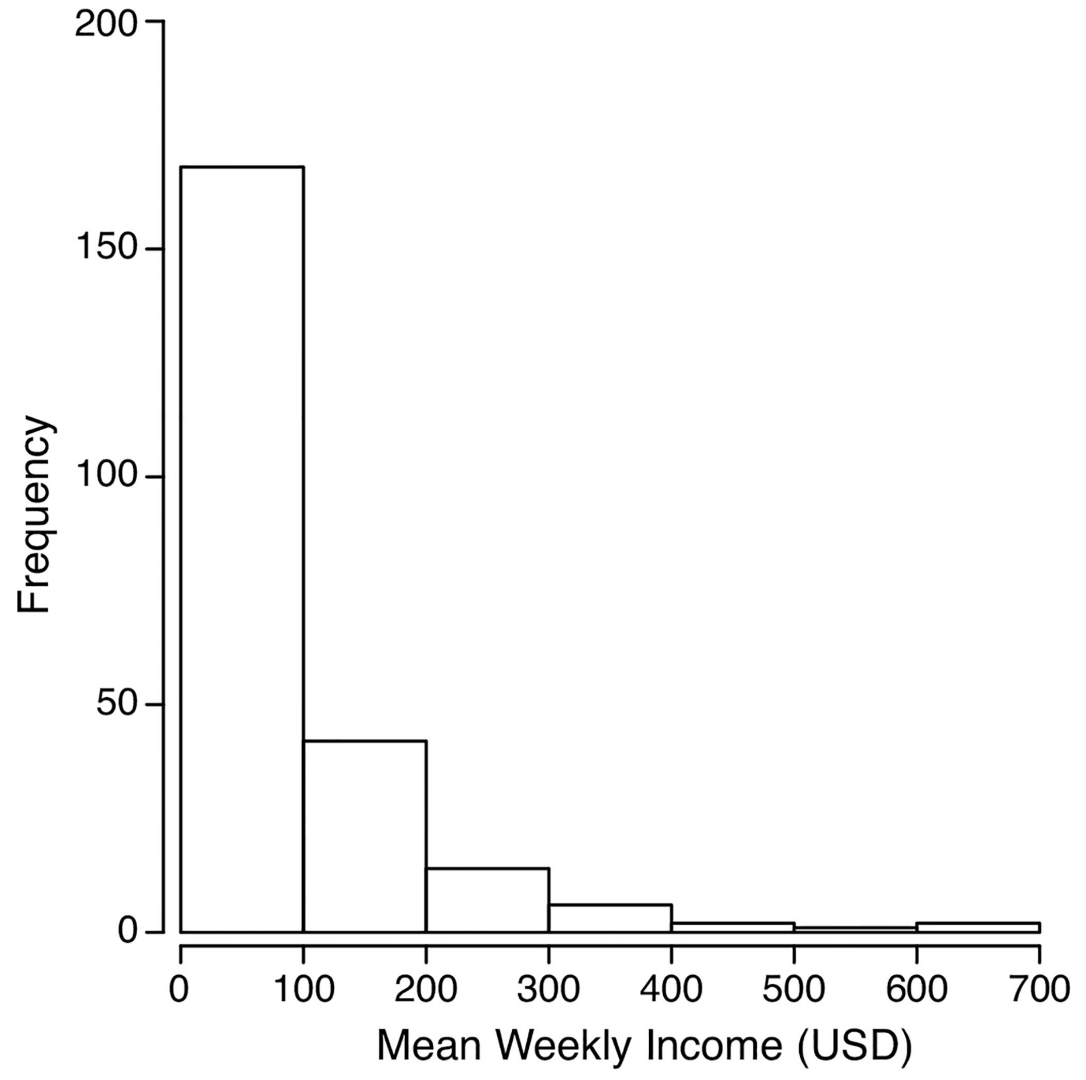
Frequency distribution of mean weekly income. Reported household mean weekly income, in USD.

There were notable differences between households in weekly income (maximum, US$700/week; median, US$63/week); however, more than 65% of the households surveyed earned US$100 or less (Fig 6), with some earning as little as US$1.40/week.

Shell money production, petty trading and fishing generally commanded the highest incomes (median weekly income >US$40 per week), while income from gardening was the lowest. For some households, gardening and fishing provided zero income, reflecting their importance for domestic consumption purposes (Table 1).

**Table 1:** Income levels for the six most common livelihood activities.

Activity	Minimum (US$/week)	Maximum (US$/week)	Median (US$/week)
**Fishing**	0.00^a^	2,142.00	43.00
**Shell money**	0.44	2,140.00	71.00
**Gardening**	0.00^a^	57.00	11.00
**Casual work**	1.40	1,700.00	14.00
**Petty trading**	1.80	770.00	45.00
**Remittance**	0.57	142.00	21.00

^a^Some households engage in fishing and gardening for food only, not to generate income, hence minimum income of $0.00 per week.

### 4.2 Dietary diversity and food insecurity

**Fig 7:**
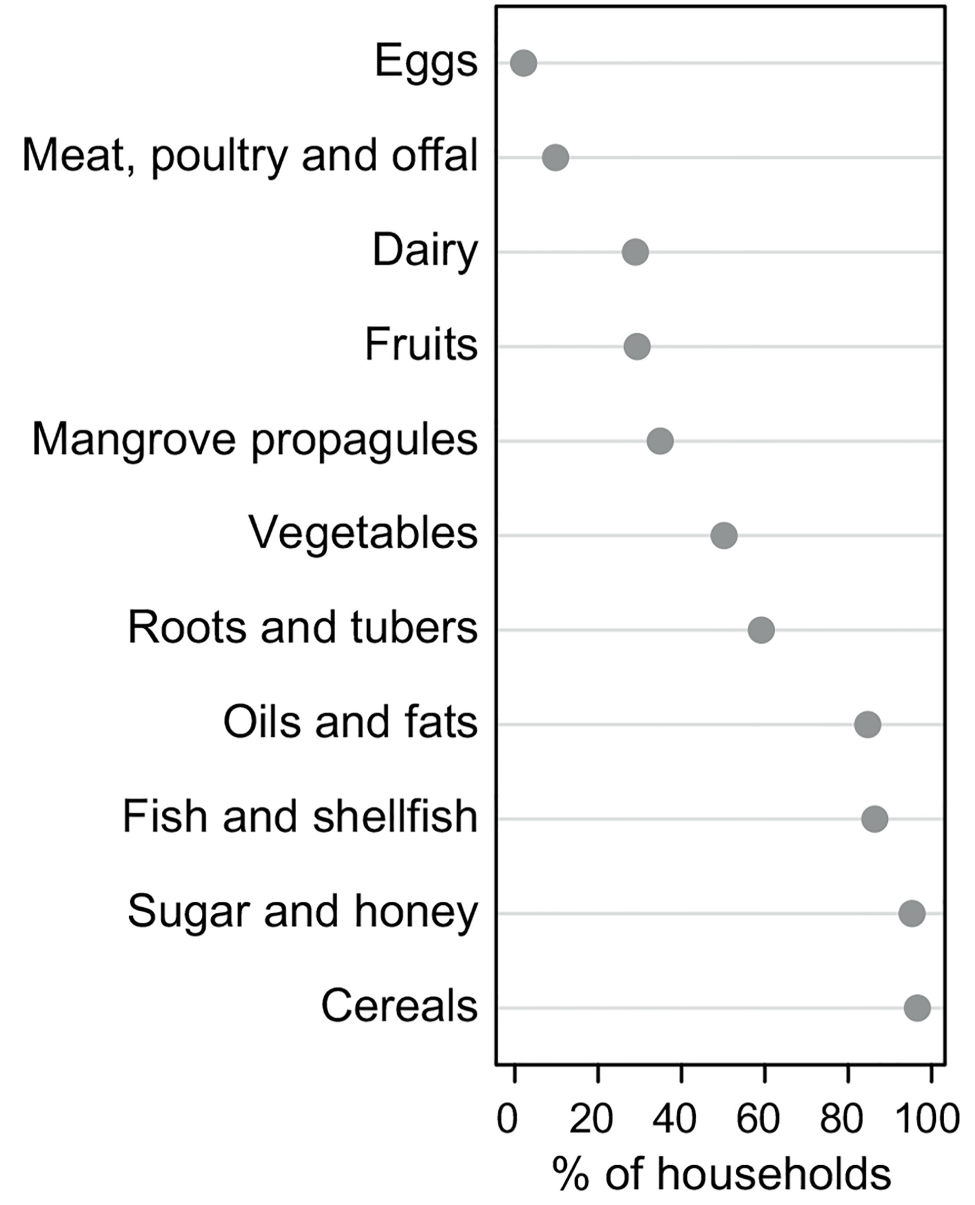
Food groups consumed by households in Langalanga lagoon. Main food groups consumed by study households in the 24 hours prior to the dietary survey.

Study households consumed between two and 10 food groups (median = 6) within the 24 hours prior to being surveyed. The most common food group was cereals bought from shops (mainly rice or flour-based products), consumed by >95% of households, followed by sugar/honey-based products (to sweeten hot beverages) (Fig 7). Fish/shellfish and oils/fats (in the form of coconut cream normally used to cook fish) had been consumed by >80% of households. Sixty per cent of surveyed households had consumed tubers, 50% had consumed other vegetables and 35% had also eaten the propagules of the mangrove species *Bruguiera gymnorrhiza*—a common food item in some parts of Solomon Islands. Around 30% of households had consumed some fruit and dairy. Less than 10% consumed meat/poultry or eggs (Fig 7). No household consumed pulses, legumes or nuts within 24 hours prior to being surveyed.

The median food insecurity score on the mainland was seven, which was not significantly different from that on the artificial islands (*U =* 6749, *p =* 0.08) where the median food insecurity score was nine. The score range for mainland households was 0–20 while on islands it was 0–26. Langalanga residents in general perceive themselves as relatively food secure. A respondent from one of the artificial islands stated the following:

*The Langalanga man although he has limited access to land is not hungry; he will make all efforts to generate income to maintain livelihoods even if it means having to go out and fish in the rain when the thunder is roaring far out in the ocean*. *The Langalanga man takes advantage of opportunities available to him*. (Translated from the original pijin)

Nevertheless there are clearly households for which this is not the case—around 10% of surveyed households returned a food insecurity score in the range of 16–26. There was no relationship between livelihood diversity and household food insecurity scores for island (*tau-b* = 0.01, *p* > 0.05) or mainland households (*tau-b* = 0.028, *p* > 0.05). There was a tendency that higher weekly income in island households makes them more food secure (*tau-b* = -0.17, *p* < 0.05). For mainland households, however, there was no significant association between weekly income and household food insecurity score (*tau-b* = 0.01, *p* > 0.05).

### 4.3 Management constituency, resource access and rule compliance

Ninety per cent of respondents stated that there was still a leader or chief in their community. These leaders were chosen either by community elections (74.0% of respondents); by the church (as pastor, catechist or other form of church leader) (20.5%); or by ‘traditional’ means (5.5%). The different categories of leaders may coexist or the same person may play different leadership roles. Sixty-four per cent of respondents agreed or strongly agreed that current community leaders or chiefs were respected, 21% said their chiefs or leaders were not respected, while 15% were unsure.

More than half of the respondents (56%) stated that the current practice of marine resource exploitation within the lagoon was an open access system in which people could fish anywhere, 34% stated that it was dictated by their village of residence with people mostly exploiting marine resources within that vicinity, while 10% stated that it was due to tribal affiliation.

Responses about resource use rules varied (Table 2). National fisheries regulations were the most frequently mentioned rules (*n* = 340), followed by traditional belief systems (*n* = 82). Misconceptions about national regulations were also common, 65 responses related to national rules which do not exist (e.g. minimum size limits on fish). Twenty five responses related to rules instituted by owners of certain spaces (e.g. reefs near tourism establishments or ‘private marine protected areas’) and 18 responses related to modern community regulations instituted under NGO-led, community-based conservation projects.

**Table 2:** Categories of different resource management rules according to type and level of compliance (%); n = number of responses given in each category (not number of respondents).

Compliance level	Gear restrictions	Species restrictions	Temporal restrictions	Size restrictions	Spatial restrictions
***Rules based on traditional beliefs (*n* = 82)***					
**All comply**	0	26	0	0	7
**Most comply**	0	24	0	0	2
**Few comply**	0	22	0	0	0
**No compliance**	0	15	0	0	4
***Rules based on privately owned areas (*n* = 25)***					
**All comply**	4	0	0	0	44
**Most comply**	0	0	0	0	20
**Few comply**	0	0	0	0	24
**No compliance**	0	0	0	0	8
***National regulations (*n* = 340)***					
**All comply**	37	2	1	0.5	0
**Most comply**	17	1.5	.5	1.5	0
**Few comply**	13	1	0	5	0
**No compliance**	17	1	0	2	0
***Misconceived regulations (*n* = 65)***					
**All comply**	3	6	0	1.5	0
**Most comply**	4.6	6	0	8	0
**Few comply**	6.1	12.3	4.6	11	1.6
**No compliance**	1.5	12.3	1.5	20	0
***Modern community regulations (*n* = 18)***					
**All comply**	0	0	0	0	5.6
**Most comply**	0	0	0	0	0
**Few comply**	0	5.6	0	5.6	22
**No compliance**	0	5.6	5.6	0	50

Responses about rules based on traditional belief systems (Table 2) included the prohibition of harvesting or consumption of certain species (sharks, rays, giant clams, crocodiles and sea cucumber) and prohibition of women entering spaces historically used in traditional religious practices. Government rules were most frequently mentioned because respondents were well informed regarding the illegal practises of dynamite and poisoning; 286 out of the 340 responses on national regulations were on dynamite and poison.

Twenty six percent of responses about rules based on traditional belief systems said that all complied with rules on species restrictions, 24% said that most comply, 22% said that few comply while 15% said that there was no compliance (Table 2). For rules on spatial restrictions, 7% of responses said that all complied, 2% said that most comply while 4% said that no one complied.

Responses about rules based on private areas were mostly spatial in nature, for example, where owners of tourism homestays prohibit the entry or use of adjacent spaces or prohibit the use of certain gears. Forty four percent of responses on spatial restrictions said that all complied, 20% said that most comply, 24% said that few comply and 8% said that there was no compliance (Table 2). Four percent of responses that identified gear restrictions also said that gear restrictions in privately owned areas were entirely complied with.

For responses on rules about national regulations, the most commonly mentioned was gear restrictions, 37% said that everyone complied, 17% said that most comply, 13% said that few comply while 17% said that no one complied with national regulations prohibiting the use of dynamite and poison for fishing (Table 2). Responses on national regulations regarding species and temporal restrictions were mostly related to the sea cucumber fishery for which there was a national ban in place at the time of the survey. For responses which stated it as a species restriction, 2% said that everyone complied, 1.5% said that most comply, 1% said that few comply and 1% said that no one complied. For responses which stated it as a temporal restriction, 1% said that it was entirely complied with and 0.5% said that most comply. For responses on size restrictions that referred to trochus size restrictions, 0.5% said that it was entirely complied with, 1.5% said that most comply with it, 5% said that few comply while 2% said that no one complied with it (Table 2).

Responses on misconceived regulations related to gear restrictions, in particular that there was a national minimum legal net mesh size. There were also misconceptions that there was a national government-imposed ban on harvest of several marine species (e.g. trochus, mangroves, some fish species and some molluscs), a size restriction for the harvest of sea cucumbers, that it was illegal to harvest small sardine-like fish and small sized scads, and that it was prohibited to enter into mangrove areas. Most of the responses on misconceived regulation also stated that few people, or no one, complied with these regulations (Table 2). The majority of responses on modern community regulations said that few people, or no one, complied with rules instituted during previous NGO-led conservation initiatives within the lagoon.

**Fig 8:**
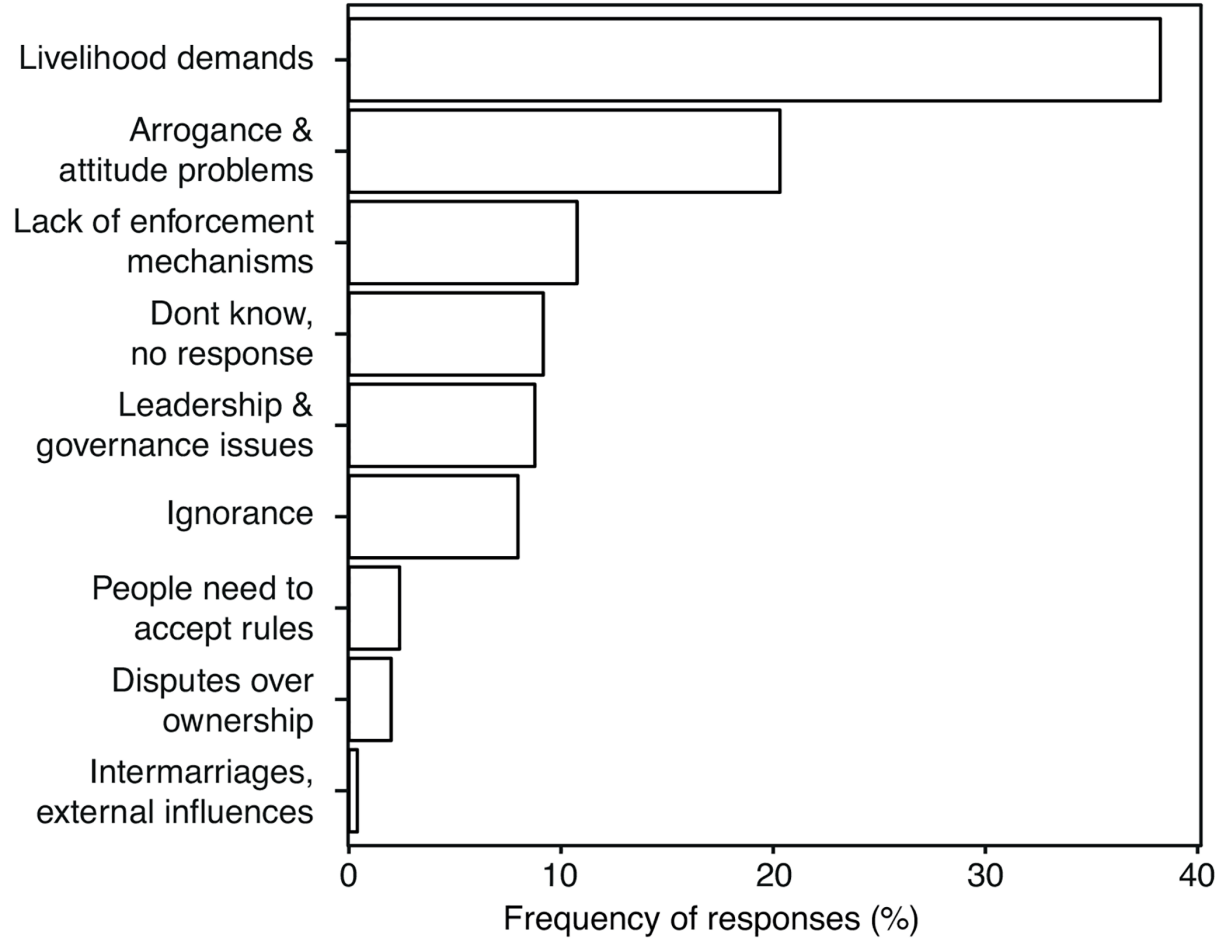
Reasons for rule violation. Categories of reasons why resource management rules were violated.

Livelihood demands (Fig 8) was the most frequent reason (38%) cited for why resource management rules were violated, followed by ‘arrogance and attitude problems’ (20%) and lack of enforcement (11%), while about 9% cited leadership and governance issues.

## Discussion

### 5.1 Livelihood diversity and the influence of proximity to an urban area

Livelihoods differed among households in the lagoon and particularly in relation to proximity to Auki. This pattern was most evident for fishing, shell money production, gardening and petty trading. Of the 30 types of livelihood activities recorded in the study, the most frequent and important were fishing, shell money production and gardening. While shell money production was only for cash income, fishing and gardening provided both food and cash income. Details of how fishing was executed varied across the lagoon. For example, although fishing within villages close to Auki was predominantly within the lagoon, some of the adult males in these villages also use outboard motor powered craft to access fishing grounds beyond the lagoon (as far as South Malaita, Nggela and Isabel) to catch mainly pelagic fish for sale at the Auki market. This was driving the trend that island households near Auki scored fishing the highest.

Langalanga is well known as the centre of shell money production in Solomon Islands [30,32,48]. While this study supports that observation, it also highlights that other livelihood activities contribute to the portfolio of livelihoods that households utilize, depending on their physical location in the environment. In addition, there was no clear relationship between livelihood diversity and income level, indicating that no single livelihood provided sufficiently high returns over the course of a year such that people don’t need to engage in other supplementary activities. Evidence from a remote island in Fiji [12] suggests that reduced livelihood diversity can result from concentration on a particular activity (carving) that generates a regular and sufficient income—in this case through the sale of carved products at the capital city, Suva. While one might postulate that shell money could play the same role in Langalanga, the supply of raw materials (various species of marine shells) can be limiting. A respondent from a village on the mainland made the following statement:

*Shells are usually not always available at the Honiara market as they are dependent on when people from western or other parts of the country may bring them to sell at Honiara; this can affect us*. *For the last several weeks I was in Honiara to buy shells but there were none available for sale immediately so I had to wait around in Honiara for 2 more weeks until shells were available for me to buy and bring home*. *During the time I was in Honiara my family was having a lot of difficulties*. *This week I have to spend more time fishing for food and to sell and get income to support our family while we work on the shells I brought*. (Translated from the original pijin language)

Clearly, other activities that also rely on natural resources, such as fishing and gardening, remain important to fall back upon when high-earning activities like shell money production have to stop temporarily due to external factors such as supply of raw material.

### 5.2 High dietary reliance on fish and diversifying strategies

Fish is the most important animal food source in the households in Langalanga Lagoon. The dietary diversity of households both on the mainland and on islands was similar, and ranged between 2 and 10 food groups. Although there was a high reliance on natural resources for food, there was evidence that store-bought foods also contribute substantially to diets, as is increasingly becoming the case in some locations in Solomon Islands [36,49] and other parts of the Pacific [50,51]. Food-insecure households had a low dietary diversity. The dietary study covered only one 24-hour recall and it is possible that seasonality [49] may play a part in availability of foods. Alternatively, maintaining a low dietary diversity could be a coping strategy for marginalised and food-insecure households, as relying on only a few basic food items may be more cost-effective in the long run than a diverse range of foods that may be more expensive [52,53], in some cases imported food items may also be more affordable than locally produced food items [54]. On the islands, households with more income were less food insecure. In contrast, there was no significant relationship between household income and food insecurity on the mainland which may be explained in part by the greater availability of land for household food production. Gardening offers a certain level of food security as indicated by the high proportion (89%) of those who garden solely for consumption. Furthermore, the lower median food insecurity score of seven observed for people settling on the mainland compared to a median score of nine for those on the islands could be attributed to food security afforded by gardening. People in these different locations utilise different strategies that are available to them as coping strategies.

We hypothesize that the poor status of the lagoon’s marine resources has driven the people of Langalanga to the highly diversified livelihood strategies observed. In that sense, the Langalanga case supports the general view that more diverse livelihoods was both a coping strategy as well as a mechanism to reduce the vulnerability of people [55–58]. Contributing to this pattern, although fishing was the most important livelihood, many other activities or livelihoods were not related to extraction of fish from the lagoon, and others were similarly uncorrelated (e.g. gardening versus shell money). This could be due to the different ways livelihoods are negotiated by different households according to the different opportunities that are available to them as per the first quotation. Livelihood diversification and any patterns thereof may be influenced by location, assets, income, varying levels of opportunities and social relations [16,56].

### 5.3 Governance institutions are no longer fit for purpose

Traditionally, and even into the early 1940s [30,32], the main form of resource access control employed in Langalanga was the establishment of closed areas, usually preceded by traditional rituals involving the sacrifice of pigs to the gods by traditional priests (*fataabu*). This enacted a conditional spell on ‘would be’ trespassers of the closed areas. A second form of access control was the gender-specific taboos that prevented women entering certain maritime spaces [30,38]. Prohibition of the consumption of certain marine species (e.g. sharks, rays, giant clams, sea cucumber) also had an unintended consequence of conserving these species. The widespread Christianisation of the area just before and after the Second World War resulted in the demise of such practices [59]. For example, although not consumed, traditionally prohibited species such as sea cucumbers and shark fins are now commonly sold to generate income. While modern Christian taboos are sometimes observed, they are mostly ignored because disregarding them is not considered as dangerous or injurious as violation of traditional taboos [30].

As populations have grown under weakening lagoon-wide governance regimes, pressure on marine resources has increased; a primary driver, as it is in so many circumstances [60,61], is the imperative to sustain livelihoods (i.e. to go fishing each day for food and income) and to harvest mangrove wood for fuel [37]. This is an important dynamic to consider for fisheries management in Langalanga Lagoon and similar locations, where dietary diversity and food insecurity scores indicate the existence of marginalised households that rely heavily on fishing. These marginalised people are usually the ones who will violate resource management rules due to the need to meet livelihood needs [61], but these marginalised people are also those with the highest stakes and have in many cases been the ones seeking assistance for managing their resources differently. Moreover, fishers with limited alternatives are likely to continue to exploit a declining fishery, reinforcing a downward-spiralling trajectory [62–64]. The net effect of these drivers of change in Langalanga is that traditional means to limit access are not fit for their modern purpose and the marine resources of the lagoon are in a parlous state.

### 5.4 Re-imagining management models in contemporary Pacific Island settings

Irrespective of whether traditional social controls were intended to limit fishing effort or manage human relations [65,66], they no longer serve as effective fishery management tools in a location such as Langalanga Lagoon. Prevalent models of CBRM which rely on clear boundaries and traditional or strong community structures [67,68] in the absence of a strong government co-management partner requires re-imagining in a location like Langalanga Lagoon where population pressure is high, an urban centre is nearby, where there still exist elements of customary ownership over the seascape, and where there is a high level of competition for resources amidst limited opportunities. A further consideration alongside livelihood pressures on resources pertains to resource management rules, which were associated, or perceived to be associated, with multiple governance actors (e.g. government, church and chiefs) and the influence of information dissemination by NGOs. While having the potential to be complementary, these can also be competitive and conflicting [69]. As evidenced in this study, the many rules associated to several governance actors appear to have resulted in a poor understanding of the status, legitimacy and purpose of the ‘rules’ people listed. Boundaries are often informal and respected by different social groups in the lagoon, which infers a dimension of rights and legitimacy that intuitively would be an opportunity for cooperating in a new and better-suited management model.

### 5.5 Navigating governance reforms in contested marine environments

Langalanga people have clearly demonstrated strength to adapt to changing circumstances and the crisis in their fisheries resources. Studies elsewhere [70] would support the recommendation that it is important that any fisheries management measure that is instituted is similarly adapted to their situation. In Lake Victoria in Kenya, Geheb and Bins [70] reported that where there was a high interdependency between fisheries and other livelihood activities, overexploitation of fisheries had resulted in some fisher families shifting to farming. State instituted fisheries management rules were established to curb fisheries overexploitation; however livelihood demands among the local populace resulted in poor compliance. It was recommended that traditional institutions which previously managed lake resources be strengthened to play a role in fisheries management. This study was prompted partially by communities expressing their desire to adapt institutions to changing conditions. Governance and resource management are evidently perceived to be important elements to enable change, but the process for reorganisation is unclear. To address environmental perturbations Armitage [17] suggested that resource management constituencies in such locations may require reorganisation or governance reform that takes into account several factors. Firstly, the need for different actors to collaboratively define problems, secondly for those actors to interact in ways that can facilitate learning and build social capital and thirdly for those actors to respond to changing patterns of marine resource use and ecological state [17]. Traditional governance/property systems still exist, and while degraded, could be reinvigorated. Such systems, together with contemporary community-based governance structures and the Provincial Government system could be the vehicle for convening the initial conversations around governance, livelihoods and resource management. External actors will likely be required to facilitate and broker conversations among communities.

Such dialogs and reorganisation should include the need to address livelihood issues and alternative livelihood options besides other issues such as governance, resource management and development aspirations. Further processes in the latter stages should involve the enactment of management institutions that enable local institutions to establish management strategies that are conscious of local household livelihood needs and are perceived as relevant and beneficial for local communities [70,71]. However, any provision of alternative livelihoods will need to be considered carefully as it has been shown that livelihood interventions, developed with an intention to serve as alternatives to reduce fishing pressure [72,73], turn out in practice to be supplements, not alternatives [74]. Governance institutions at scales larger than that of the community bring new complexities and opportunities for people to be excluded from decision-making. During such governance transitions, there is a strong imperative to hear the voices of marginalized people and to ensure their access to benefits that flow from improved governance; doing so will accelerate development.

## Conclusion

In this paper we have examined livelihoods and fisheries governance in a location where there is a high reliance on fisheries amidst limited land-based alternatives. We showed that livelihoods are negotiated according to different opportunities that are available including markets and land-based natural resources. The imperative for food and income from fisheries coupled with weakening traditional management systems presents challenges for fisheries governance and management in such locations. Fisheries management will require not only commitment from local stakeholders but also the involvement of local governance institutions and other actors such as NGO’s to broker negotiations between different local stakeholders. Navigating new approaches to fisheries governance and management must take into account existing livelihood strategies if they are to succeed.
